# Developmental screening tools for identification of children with developmental difficulties in high-income countries: a systematic review

**DOI:** 10.3389/frcha.2023.1074004

**Published:** 2023-07-06

**Authors:** Sara Cibralic, Patrick Hawker, Ferosa Khan, Abbie Lucien, Antonio Mendoza Diaz, Susan Woolfenden, Elisabeth Murphy, April Deering, Clare Schnelle, Sharnee Townsend, Valsamma Eapen

**Affiliations:** ^1^ Ingham Institute, Liverpool, NSW, Australia; ^2^ Psychiatry and Mental Health, School of Clinical Medicine, University of New South Wales, Randwick, NSW, Australia; ^3^ Sydney Local Health District, Sydney Institute Women, Children and Their Families, Camperdown, NSW, Australia; ^4^ New South Wales Ministry of Health, St Leonards, NSW, Australia; ^5^ Academic Unit of Infant Child and Adolescent Services (AUCS), SWSLHD, Liverpool, NSW, Australia

**Keywords:** developmental screening, developmental surveillance, tools, young children, review, high-income countries

## Abstract

**Objective:**

To examine and synthesize the literature on the use of universal developmental screening and surveillance tools in high-income countries in relation to (1) psychometric properties; (2) knowledge, acceptability, and feasibility of tools; and (3) follow-up taken following screening/surveillance.

**Method:**

A PRISMA-compliant systematic review was performed in the PsychInfo, PubMed, and Embase databases. Studies published in the English language were included if they reported results evaluating a universal developmental screening or surveillance measurement tool. Articles on service providers’ and/or parents’ views on developmental screening were also included. Two independent reviewers extracted data and assessed for risk of bias using the Mixed Methods Appraisal Tool and the Quality Assessment of Diagnostic Accuracy Studies Tool. Results were synthesized qualitatively.

**Results:**

Initial searches identified 2,078 articles, of which 52 were included in the final review. Findings showed that several articles assessing the accuracy of screening tools have been published, and together, they suggest that the accuracy of screening tools varies across cultures and countries. Furthermore, available literature indicated that administering universal developmental screening tools was feasible and acceptable, though only a small number of studies are available. Results also showed a limited number of studies looking at actions taken following positive screening results.

**Conclusion:**

As the evidence stands, more research assessing the acceptability, feasibility, and accuracy of developmental screeners, is needed.

**Systematic review registration:**

This review has been registered with the University of York Centre for Reviews and Dissemination (PROSPERO; https://www.crd.york.ac.uk/prospero/display_record.php?RecordID=337320, registration number CRD42022337320).

## Introduction

Developmental difficulties detected during childhood, such as intellectual disorders, communication disorders, autism spectrum disorder, attention deficit/hyperactive disorder, and specific learning disorders, are prevalent (1, 2). When developmental difficulties persist, especially within environments that do not cater to developmental difficulties (3), they can be associated with a range of negative outcomes, including emotional (4, 5) and behavioural (6) problems. Early detection and intervention for developmental difficulties results in the best outcomes for children (7–10). It has therefore been recommended by policy bodies that universal developmental surveillance and screening is undertaken with all children under 5 years of age (11).

Developmental screening, which refers to the use of standardised instruments to aid in the identification of developmental difficulties at a specific point in time (12, 13), has been found to increase early identification of developmental difficulties, diagnosis, and access to early intervention (14, 15). Developmental screening forms part of the developmental surveillance process (12), which refers to the ongoing clinical monitoring of children at risk of developmental difficulties (12, 16). Additional components of developmental surveillance include observing children during healthcare appointments, discussing caregivers’ concerns, obtaining a child's developmental history from caregivers, and sharing any concerns with other health professionals (12). Developmental screening does not result in a diagnosis, but it can increase the sensitivity and specificity of surveillance outcomes such as an accurate diagnosis (8, 14, 15). Developmental screening was originally recommended by the American Academy of Paediatrics in 2001 (12). In 2006 The American Academy of Pediatrics developed The Brighter Futures guidelines for health supervision (17), in which the Council on Children with Disabilities (18) recommended developmental surveillance at routine well-child visits, developmental screening at specific ages (e.g., 9, 18, and 30 months) or when surveillance indicates that screening is needed (18). Subsequent research has shown that developmental surveillance together with developmental screening, compared to developmental surveillance alone, results in greater identification of delays, referrals to early intervention, and access to early intervention (14, 19). A randomized control trial in the United States of America (USA) (14), for example, found that when young children (*N* = 2,104, aged <30 months) were screened with the Ages and Stages Questionnaire-II and Modified Checklist for Autism in Toddlers with office staff assistance or without office staff assistance were 23.0% and 26.8%, respectively, more likely to be identified with delays compared 13.0% of children who received developmental surveillance alone.

Since the introduction of developmental screening into the developmental surveillance process, advances in our understanding of child development have resulted in the creation of numerous developmental screening and surveillance tools, and several systematic reviews and reports evaluating these tools have been undertaken (20–25). The majority of available reviews have however focused on the use of developmental surveillance and screening tools in low-to-middle-income countries (e.g., 21). Therefore, this review aimed to synthesise and evaluate the literature on the use of developmental screening and surveillance tools in high-income countries (see Supplementary Tables S2, S3 for a definition of high-income countries and a list of high-income countries as of 2021).

The review's objectives were:

1. Identify literature on developmental screening and surveillance tools used with children aged 0–5 years in high-income countries.
2. Report on the psychometric properties of identified screening tools.
3. Determine the knowledge, acceptability (parental and service provider) and feasibility of identified developmental screening and surveillance tools.
4. Identify whether referrals were implemented, and follow-up undertaken for children following screening.

## Methods

Prior to the commencement of this review, a study protocol was developed and registered with the University of York Centre for Reviews and Dissemination (PROSPERO; registration number: CRD42022337320).

### Search strategy

A systematic search of published literature available up to May 2022 was conducted (no limits were placed on the earliest possible starting date). Four search strategies were implemented to identify relevant research studies. First, interdisciplinary research databases PsychInfo, Embase, and PubMed were searched concurrently for entries containing any of the following search terms: “child” OR “infant*” OR “baby” OR “preschool” AND “milestone*” AND “surveillance” OR “screening tool*” OR “screening measure*” OR “screening assessment*”. All searches were limited to entries conducted on “human” subjects published in an “English Language” journal. Second, the reference lists of articles selected for this review were searched manually. Third, internet searches for grey literature were conducted using the above-mentioned search terms alongside focused searches on key websites, including screening tool developer websites. Fourth, the “cited by” option available on some databases was used to manually search articles that had referenced the articles selected for this review. As per Preferred Reporting Items for Systematic Reviews and Meta-Analysis (PRISMA) guidelines (26), Supplementary Table S1 provides an example of the search strategy approach.

### Inclusion and exclusion criteria

Articles were included for full text review if: (1) they evaluated a universal developmental screening or surveillance tool which included greater than two developmental domains (i.e., studies on screeners focused only on one domain such as gross motor were excluded); (2) the study sample included participants aged 0–5 years (if samples comprised a wide age group they were included if the average child age was below 6 years); (3) the study was undertaken in a high-income country; and (4) the article was published in English. Articles that looked at practitioner and parent acceptability of screening/surveillance tools were also included if they were published in English. Articles were excluded if: (1) they included a screening/surveillance tool to evaluate an intervention outcome only (i.e., there was no evaluation of the screening tool itself); (2) they used a screening/surveillance tool to evaluate development in a specific population only (e.g., children with congenital heart disease) or focused on a specific condition (e.g., autism spectrum disorder only); (3) they were not available in English; (4) they were not data-based (e.g., books, theoretical papers, reviews); or (5) they were unpublished dissertations/theses. Articles focused on First Nations populations were also excluded as a separate review on developmental screening/surveillance tools used with First Nations populations was undertaken (27). Articles that focused on a specific population but had a comparison group drawn from a normative population and segregated data were included, however, only data on the comparison group was interpreted. Furthermore, studies that included results from multiple countries were only included if data was segregated based on country and results from high-income countries were interpretable.

### Screening

Four reviewers independently performed title/abstract screening and full-text screening (two reviewers focused on articles published prior to 2014 (team 1) and two reviewers (team 2) focused on articles published after 2014). In cases where there were disagreements between the reviewers, they resolved these through further discussion. A third reviewer was available to settle conflicts if necessary. The inter-rater reliability for title/abstract screening was 88% and 83% for team 1 and team 2, respectively. The inter-rater reliability for full text screaming was 97% for team 1% and 89% for team 2.

### Quality assessment and data analysis

Two reviewers independently assessed the quality of studies using the Mixed Methods Appraisal Tool [MMAT; (28)] or the Quality Assessment of Diagnostic Accuracy Studies Tool [QUADAS-2; (29)]. The MMAT allows for the assessment of the methodological quality of qualitative research, randomized controlled trials, non-randomized studies, quantitative descriptive studies, and mixed methods studies. Using MMAT, the risk of bias is determined based on five sources for each study category. For example, for randomized control trials, study quality is based on (1) randomization; (2) group comparability; (3) complete outcome data; (4) blinding of assessors; and (5) participant intervention adherence. Reviewers first ascertained the study design by evaluating the methodology using the MMAT. They then assigned a “yes”, “no”, or “can't tell” to each outcome. The “can't tell” option was used when not enough information is available for the reviewer to assign a “yes” or “no”. Disagreements were discussed between reviewers, and a third reviewer was available to settle discrepancies, ensuring a consistent and reliable assessment of study quality. The MMAT is however not suitable for use with diagnostic accuracy studies. Therefore, the QUADAS-2 (29) was used to evaluate diagnostic accuracy studies. The risk of bias on the QUADAS-2 is determined based on (1) patient selection; (2) index text; (3) reference standard; and (4) flow and timing. The QUADAS-2 also allows you to assess applicability based on concerns that the study does not match the review questions. For each of the four domains in QUADAS-2 we followed a structured approach that involved assessing signalling questions, making judgments about the risk of bias, and evaluating applicability concerns. Risk of bias was judged as “low,” “high,” or “unclear.” If the answers to all signalling questions for a domain were “yes,” then the risk of bias for that domain was judged as “low”. If any signalling question was answered “no” or “unclear”, potential for bias was denoted as “high”, or “unclear”, respectively. The same criteria were applied to assess applicability concerns, ensuring a comprehensive evaluation of each study's relevance to our review questions. Any discrepancies in the overall risk of bias or applicability judgments (for each of the four domains) were discussed between the reviewers and resolved through agreement. A third reviewer was available to settle disputes if necessary. As both the MMAT and QUADAS-2 discourage the calculation of an overall quality score an overall quality score was not calculated (see Tables 1, 2 for quality assessments of the included studies).

**Table 1:** Quality assessment using QUADAS-2.

Citation	Risk of bias				Applicability concerns		
	Patient selection	Index test	Reference standard	Flow and timing	Patient selection	Index test	Reference standard
Brothers et al. (30)	L	?	L	?	L	L	L
Clark et al. (31)	L	L	L	H	L	L	L
Deakin-Bell et al. (32)	?	?	?	L	L	L	L
Dixon et al. (33)	H	?	?	L	L	L	L
Glascoe et al. (34)	L	L	L	?	?	L	L
Glascoe and Byrne (35)	L	L	L	?	?	L	L
Gollenberg et al. (36)	L	L	?	L	L	L	L
Hatakenaka et al. (37)	?	?	H	H	L	L	L
Hatakenaka et al. (38)	?	L	H	H	L	L	L
Hess et al. (39)	L	?	?	H	L	L	L
Kenny et al. (40)	L	L	?	H	L	L	L
King-Dowling et al. (41)	L	?	?	L	L	L	L
Krijnen et al. (42)	L	L	?	?	L	L	L
Li et al. (43)	L	?	?	H	L	?	L
Limbos et al. (44)	H	L	L	L	L	L	L
Mezawa et al. (45)	L	L	L	?	L	L	L
Rydz et al. (46)	L	L	L	H	L	L	L
Schonhaut et al. (47)	?	L	?	L	L	L	L
Sheldrick et al. (48)	L	L	L	?	L	L	L
Squires et al. (49)	L	L	?	H	L	L	L
Squires et al. (50)	L	L	?	H	L	L	L
Tveten et al. (51)	L	L	L	L	L	L	L
Williams et al. (52)	L	L	H	L	H	L	L
Yue et al. (53)	?	?	?	?	L	L	L

L, low; H, high; ?, unclear.

**Table 2:** Quality assessment using MMAT.

Citation			Criteria		
Qualitative	1.1. Is the qualitative approach appropriate to answer the research question?	1.2. Are the qualitative data collection methods adequate to address the research question?	1.3. Are the findings adequately derived from the data?	1.4. Is the interpretation of results sufficiently substantiated by data?	1.5. Is there coherence between qualitative data sources, collection, analysis and interpretation?
Garg et al. (54)	Y	Y	Y	Y	Y
Raspa et al. (55)	Y	Y	Y	Y	Y
Quantitative nonrandomized	3.1. Are the participants representative of the target population?	3.2. Are measurements appropriate regarding both the outcome and intervention (or exposure)?	3.3. Are there complete outcome data?	3.4. Are the confounders accounted for in the design and analysis?	3.5. During the study period, is the intervention administered (or exposure occurred) as intended?
Berger-Jenkins et al. (56)	Y	Y	Y	Y	Y
Chen et al. (57)	N	Y	?	?	Y
Coghlan et al. (58)	N	Y	Y	Y	Y
Dean et al. (59)	N	Y	?	?	Y
Elbers et al. (60)	Y	Y	Y	Y	Y
Hatakenaka et al. 2019	Y	Y	Y	?	Y
Heo et al. (61)	Y	Y	Y	Y	Y
Janson et al. (62)	N	Y	Y	?	Y
Kiing et al. (63)	?	?	Y	Y	Y
Kwan and Nam (64)	N	Y	N	Y	Y
Lamsal et al. (65)	Y	Y	Y	Y	Y
Lopes et al. (66)	Y	Y	Y	Y	Y
Lung et al. (67)	Y	Y	Y	Y	Y
Olvera Astivia et al. (68)	Y	Y	N	Y	Y
Sarmiento Campos et al. (69)	Y	Y	Y	Y	Y
Sices et al. (70)	Y	Y	N	Y	Y
Tsai et al. (71)	?	Y	Y	?	Y
Williams et al. (72)	N	Y	Y	Y	Y
Quantitative descriptive	4.1. Is the sampling strategy relevant to address the research question?	4.2. Is the sample representative of the target population?	4.3. Are the measurements appropriate?	4.4. Is the risk of nonresponse bias low?	4.5. Is the statistical analysis appropriate to answer the research question?
Sheldrick et al. (73)	Y	Y	Y	?	Y
Mixed methods	5.1. Is there an adequate rationale for using a mixed methods design to address the research question?	5.2. Are the different components of the study effectively integrated to answer the research question?	5.3. Are the outputs of the integration of qualitative and quantitative components adequately interpreted?	5.4. Are divergences and inconsistencies between quantitative and qualitative results adequately addressed?	5.5. Do the different components of the study adhere to the quality criteria of each tradition of the methods involved?
Abercrombie et al. (74)	Y	Y	Y	Y	N
Cheng et al. (75)	Y	Y	?	?	N
Cheng et al. (76)	Y	Y	?	?	N
Dixon et al. (33)	Y	Y	Y	Y	N
Gadomski et al. (77)	Y	Y	Y	Y	Y
Graybill et al. (78)	Y	Y	Y	Y	Y
Taylor et al. (79)	Y	Y	Y	Y	N

All studies met MMAT screening questions criteria S1, “Are there clear research questions?”; and S2, “Do the collected data allow to address the research questions?”. Y, yes; N, no; ?, can't tell.

## Results

**Figure 1:**
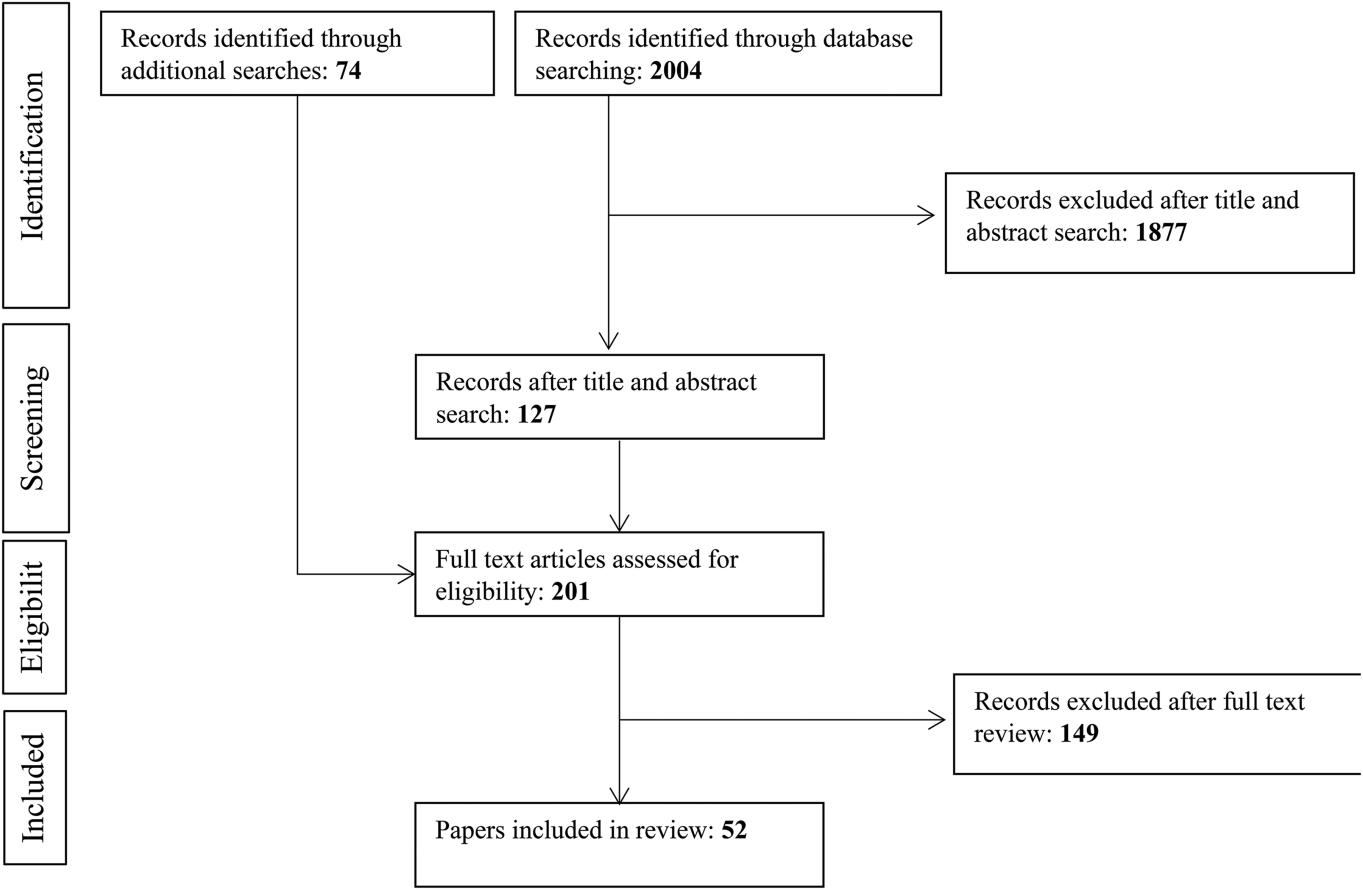
PRISMA flow diagram of included studies.

Figure 1 presents an overview of our search strategy. Initial database searches resulted in a total of 2004 articles. Title and abstract screening resulted in the exclusion of 1,877 articles, resulting in 127 articles. Seventy-four additional articles were identified through reference lists, cited by, and grey literature searches, resulting in a total of 201 articles that underwent full-text screening. Full-text screening resulted in the exclusion of an additional 149 articles. Fifty-two articles met the inclusion criteria and were included in the current review.

### Overview of included studies

Fifty-two studies evaluating universal developmental surveillance or screening tools were identified, Table 3 presents an overview of studies included in this review. Of those, 22 were undertaken in the USA, six in Canada, four in Japan, six in China, four in Australia, two in Norway, two in Singapore, and one each in Chile, Korea, The Netherlands, Portugal, Spain, and Wales. Thirty-two were cross-sectional, nine were longitudinal, six were mixed methods, three were qualitative, one was a randomized control trial, and one was a prospective population-based observational study. Most tools were universal screening tools, apart from *Learn the Signs. Act Early* which is a surveillance tool.

**Table 3:** Overview of included studies table.

Citation	Study classification	Country	Recruitment site	Person who completed tool	Demographics	Psychometric properties assessed	Knowledge, feasibility, and/or acceptability of tool assessed	Actions taken after tool was administered
ASQ (24 studies)								
Chen et al. (57)	Cross-sectional	United States of America	Community	Not specified	*N* = 1,691; child age range = 15 months 0 days to 16 months 30 days; young children; 58% male	Y	N	N
Deakin-Bell et al. (32)	Cross-sectional	Australia	Primary care^a^	Parents	*N* = 166 control children; child age = 12 months; sex not provided	Y	N	N
Elbers et al. (60)	Longitudinal	Canada	Primary care	Parents	*N* = 68 children from the community; age range = 4–36 months; sex not provided	Y	N	Y
Garg et al. (54)	Qualitative	Australia	Primary care	Healthcare staff	*N* = 37 health professionals. 24.3% male. 22 nurses, 8 paediatricians, 6 GPs, and 1 allied health worker	N	Y	N
Gollenberg et al. (36)	Longitudinal	United States of America	Primary care	Parents/primary caregivers	*N* = 40; child age = 24 months; 48% male	Y	N	N
Heo et al. (61)	Cross-sectional	Korea	Community and health care settings	Parents	*N* = 3,220; child age range = 4–60 months. Male = 50.3%	Y	N	N
Janson et al. (62)	Longitudinal	Nrway	Community	Parents/caregivers	*N* = 1,340; child age range = 4 months to 5 years; sex not provided	Y	N	N
King-Dowling et al. (41)	Cross-sectional	Canada	Community	Primary caregivers or parents	*N* = 159 children. Age range 43–65 months; 49% male	Y	N	N
Krijnen et al. (42)	Cross-sectional	Netherlands	Community	Parents	*N* = 1,014; 93.9% = health children and 4.3% = prematurely born children, 1.8% = children with other risk factors; child age range = 3–41 months; 50.7% male	Y	N	N
Lamsal et al. (65)	Prospective longitudinal	Canada	National survey	Person most kNwledgeable about the child	*N* = 17,746; child age range 4–5 years; 65.75% male	Y	N	N
Limbos et al. (44)	Cross-sectional	Canada	Primary care	Parents	*N* = 334 children; age range = 12–60 months; 56% male	Y	N	Y
Lopes et al. (66)	Cross-sectional	Portugal	Primary care or childcare	Primary caregivers or parents	*N* = 234; child age range 9–30 months, 52.6% male	Y	N	N
Mezawa et al. (45)	Cross-sectional	Japan	Secondary care	Parents	*N* = 439; child age range 6–60 months; 52% male	Y	N	N
Olvera Astivia et al. (68)	Longitudinal	United States of America	Child development support program	Parents	*N* = 5,056; child range = 12–48 months; sex not provided	Y	N	N
Rydz et al. (46)	Cross-sectional	Canada	Primary care	Parents	*N* = 248 (*n* = 134 ASQ); child age range = 17.03–20.47 months; 53% male	Y	N	N
Sarmiento Campos et al. (69)	Cross-sectional	Spain	Pre-Primary school	Educators	*N* = 1,089 families; child age range 6–42 months; sex of entire sample not provided	Y	N	N
Schonhaut et al. (47)	Cross-sectional	Chile	Primary care	Parents	*N* = 306 (119 = born term); 110 = 8 months (59% male); 100 = 18 months (53% male); 96 = 30 months (45% male)	Y	N	N
Sheldrick et al. (48)	Cross-sectional	United States of America	Primary care	Parents	*N* = 1,495 children. Age range 9 months to 5.5 years, mean age (SD) 2.6 (1.3) years, 52.1% male.	Y	N	N
Sices et al. (70)	Cross-sectional	United States of America	Primary care	Parents	*N* = 60; child age range = 9–31 months; 58 male	Y	N	N
Squires et al. (50)	Cross-sectional	United States of America	Community	Parents/caregivers	*N* = 2,008; child age range = 4–48 months; 53% male	Y	N	N
Squires et al. (49)	Longitudinal	United States of America	Community and health care settings	Parents	*N* = 96; child age range = 4–30 months; sex not provided	Y	N	N
Tsai et al. (71)	Cross-sectional	China	Preschools	Parents and teachers	*N* = 112; child age range = 34–38 months; 55% male	Y	N	N
Tveten et al. (51)	Cross-sectional	Nrway	Primary care	Parent	*N* = 432; child age range = 3–12 months; 52% male	Y	N	N
Yue et al. (53)	Cross-sectional	China	Community	Trained staff	*N* = 1,831; child age range 5–24 months, 52% male	Y	N	N
PEDS (9 studies)								
Brothers et al. (30)	Cross-sectional	United States of America	Community, primary care, schools	Unclear	*N* = 1,619; child age range = 0–95 months; 51% male	Y	N	N
Coghlan et al. (58)	Cross-sectional	Australia	Day care and kindergarten centres	Parents	*N* = 262; child age range 18 months to 5 years, 9 months; 47% male	N	Y	Y
Cox et al. (80)	Mixed methods	United States of America	Primary care	Parents	*N* = 752; child age range = 6 months to 9 years; 49% male	Y	N	N
Garg et al. (54)	Qualitative	Australia	Primary care	Healthcare staff	*N* = 37 health professionals. 24.3% male. 22 nurses, 8 paediatricians, 6 GPs, and 1 allied health worker.	N	Y	N
Kiing et al. (63)	Cross-sectional	Singapore	Kindergarten and childcare centres	Parents, teachers, childcare workers	*N* = 1,806 children; child age range = 1 month to 6 years, 11 months; 54% male	Y	N	N
Limbos et al. (44)	Cross-sectional	Canada	Primary care	Parents	*N* = 334 children; age range = 12–60 months; 56% male	Y	N	Y
Sarmiento Campos et al. (69)	Cross-sectional	Spain	Pre-Primary school	Educators	*N* = 1,089 families; child age range 6–42 months	Y	N	Y
Sheldrick et al. (48)	Cross-sectional	United States of America	Primary care	Parents	*N* = 1,495 children. Age range 9 months to 5.5 years, mean age (SD) 2.6 (1.3) years, 52.1% male.	Y	N	N
Sices et al. (70)	Cross-sectional study.	United States of America	Primary care	Parents	*N* = 60; child age range = 9 –31 months; 58% male	Y	N	N
LTSAE (5 studies)								
Abercrombie et al. (74)	Mixed methods	United States of America	Childcare	Parents and staff	*N* = 487 parents, staff, and managers.	N	Y	Y
Gadomski et al. (77)	Mixed methods	United States of America	Primary care	Parents	*N* = 181; Child age range = 2 months to 3 years; sex not provided	N	Y	Y
Graybill et al. (78)	Mixed methods	United States of America	Primary care	Parents	*N* = 108; Child age range 0–5 years, sex not provided. Parents age range = 20–59 years, 10% male	N	Y	Y
Raspa et al. (55)	Qualitative	United States of America	Childcare	Parents	Focus group: *N* = 74 parents, 4% male; In-Depth interview: *N* = 21 parents, 24% male	N	Y	Y
Taylor et al. (79)	Mixed methods	United States of America	Childcare	Parents, staff, and leadership	Parents: *N* = 125, 22% male; Staff: *N* = 30, 0% male; Leadership: *N* = 15, 0% male.	N	Y	N
SWYC (3 studies)								
Berger-Jenkins et al. (56)	Cross-sectional	United States of America	Primary care	Parents or primary caregivers	*N* = 349; Child age range 6 months to 10 years; 53% male	N	Y	Y
Sheldrick et al. (73)	Cross-sectional	United States of America	Primary care	Parents	*N* = 13,076 children; Rhode Island Cohort: 51% male; Minnesota Cohort: 52% male; Massachusetts Cohort: 52.4% male	Y	Y	N
Sheldrick et al. (48)	Cross-sectional	United States of America	Primary care	Parents	*N* = 1,495; child age range = 9 months to 5.5 years; 52.1% male.	Y	N	N
ESSENCE-Q (3 studies)								
Hatakenaka et al. (38)	Prospective population-based observational	Japan	Primary care	Parents and trained staff	Cohort 1: *N* = 152 children at 18-months check-ups; child age range 18–21 months; 52.4% male. Cohort 2: *N* = 149 children at 36-months check-ups. Age range 41–43 months, mean age (SD) 42.0 (1.0) months, 49% male.	Y	N	Y
Hatakenaka et al. (37)	Cross-sectional	Japan	Secondary care	Primary caregivers or parents	*N* = 130 children; child age range = 21–72 months; 84% male.	Y	N	N
Hatakenaka et al. (81)	Cross-sectional	Japan	Primary care	Parents	*N* = 207; child age = 20-month check-up (56% male), 40 month check-up (49% male)	Y	N	Y
Taipei (2 studies)								
Cheng et al. (75)	Mixed methods	China	Primary care	Primary caregivers	*N* = 390; child age range 4–84 months; sex not provided	Y	N	N
Cheng et al. (76)	Mixed methods	China	Primary care	Primary caregivers	*N* = 120; child age range = 4–30 months; sex not provided	N	Y	N
Denver- II (2 studies)								
Glascoe et al. (34)	Cross-sectional	United States of America	Childcare centres	Psychologist	*N* = 104; child age range = 3–72 months; 50% male	Y	N	N
Glascoe and Byrne (35)	Cross-sectional	United States of America	Childcare centres	Psychologist	*N* = 89; Child age range = 7–70 months. 52% male	Y	N	N
BINS (1 study)								
Hess et al. (39)	Longitudinal	United States of America	Secondary care	Research assistant	*N* = 106, child age range = 6–36 months; sex not provided	Y	N	Y
DSQ (1 study)								
Kwan and Nam (64)	Cross-sectional	Singapore	Community and primary care	Parents	*N* = 506; child age range 6–78 months. 50.3% male	Y	N	N
CREDI (1 study)								
Li et al. (43)	Randomized control trial	China	Community	Caregiver	*N* = 946; child age range = 5–35 months; 48.5% male	Y	N	N
CDI (1 study)								
Rydz et al. (46)	Cross-sectional	Canada	Primary care	Parents	*N* = 248 (*n* = 114 CDI); child age range = 17.03–20.47 months; 53% male	Y	N	N
IMQ (1 study)								
Dixon et al. (33)	Longitudinal	Australia	Secondary care	Parents	*n* = 347 (control infants); child age at assessment = 4, 8, 12, 18, 24, 36, and 48 months; 54% male	N	N	N
EYCI (1 study)								
Clark et al. (31)	Cross-sectional	Canada	Childcare	Parents	*N* = 246: *n* = 47 in phase 1, *n* = 199 phase 2; child age range = 18–42 months; 51% male	Y	N	N
MPI (1 study)								
Williams et al. (72)	Cross-sectional	United States of America	Kindergarten	Teachers aids and volunteers	*N* = 40; child age range = 55–72 months; 32.5% male.	Y	N	N
MSCA (1 study)								
Dean and Steffen (59)	Cross-sectional	United States of America	Nursery-school program	Parents	*N* = 86; child age range = 2.6–5 years; 48% male.	Y	N	N
MCDI (1 study)								
Kenny et al. (40)	Cross-sectional	United States of America	Longitudinal birth cohort study participants	Parents	*N* = 364; child age = 3 years; sex not provided	Y	N	Y
SGS-II (1 study)								
Williams et al. (52)	Cross-sectional	Wales	Nursery and nursery schools	Post-graduate student	*N* = 43; age range = 0–5 years; 51% males	Y	N	Y
TBCS (1 study)								
Lung et al. (67)	Longitudinal	China	Community	Parents	*N* = 1,630; child age = 36 months; 55.2% male	Y	N	N

Y, yes (included in study); N, no (not included in study). ASQ, ages and stages questionnaire; BINS, Bayley infant neurodevelopmental screener—II; CDI, child development inventory; CREDI, caregiver reported early development instruments; Denver II, Denver developmental screening test; DSQ, developmental screening questionnaire; ESSENCE-Q, early symptomatic syndromes eliciting neurodevelopmental clinical examinations-questionnaire; EYCI, early years check-in; IMQ, infant/child monitoring questionnaire; LTSAE, learn the signs act early; MCDI, Minnesota child development inventory; MPI, the Minnesota preschool inventory individual progress-shortened form; MSCA, McCarthy scale of children's abilities—short from; PEDS, parents evaluation of developmental status; SGS-II, the schedule of growing skills II; SWYC, survey of wellbeing of young children; Taipei II, Taipei City child development screening tool, second version; TBCS, the Taiwan birth cohort study.

^a^ Primary care setting refers to general practitioner and community health settings while secondary care setting refers to hospital and specialist settings.

Twenty four studies evaluated the Ages and Stages Questionnaire [ASQ; (32, 36, 41, 42, 44–51, 53, 54, 57, 60–62, 65, 66, 68–71)] and one study evaluated the Infant/Child Monitoring Questionnaire [IMQ; (33)] which was later renamed the ASQ; nine studies evaluated the Parents Evaluation of Developmental Status [PEDS; (30, 44, 48, 54, 58, 63, 69, 70, 80)]; five studies evaluated the Learn the Signs. Act Early [LTSAE; (55, 74, 77–79)]; three studies evaluated the Survey of Wellbeing of Young Children [SWYC; (48, 56, 73)]; three studies evaluated the Early Symptomatic Syndromes Eliciting Neurodevelopmental Clinical Examinations-Questionnaire [ESSENCE-Q; (37, 38, 81)]; two studies evaluated the Denver Developmental Screening Test [Denver II; (34, 35)]; two studies evaluated the Taipei City Child Development Screening tool, second version [Taipei II; (75, 76)]; and the Bayley Infant Neurodevelopmental Screener—II [BINS; (39)], Child Development Inventory [CDI; (46)], Caregiver Reported Early Development Instruments [CREDI; (43)], Developmental Screening Questionnaire [DSQ; (64)], Early Years Check-In [EYCI; (31)]; Minnesota Child Development Inventory [MCDI; (40)], The Minnesota Preschool Inventory Individual Progress-Shortened Form [MPI; (72)], McCarthy Scale of Children's Abilities—Short From [MSCA; (59)], The Schedule of Growing Skills II [SGS-II; (52)], and The Taiwan Birth Cohort Study [TBCS; (67)] were all evaluated in one study only.

Forty-three studies assessed the psychometric properties of a developmental screening/surveillance tool (30–53, 57, 59–73, 75, 78, 80, 81), nine evaluated the knowledge, acceptability, and/or feasibility of a developmental screening/surveillance tool (54–56, 58, 74, 76–79), and nine reported on actions taken following screening (38, 40, 44, 56, 58, 60, 69, 74, 81).

### Psychometric properties of identified developmental screening tool

Prior to providing an overview of the psychometric properties of identified tools, it is important to note that of the identified studies which looked at the sensitivity and specificity of screeners, most studies operationalised sensitivity and specificity as the correlation between the screener score and the score on a validated measure of developmental status such as Bayley Scale of Infant and Toddler Development [e.g., (32, 36)]. Other identified studies operationalised sensitivity and specificity as the correlation between screener scores, and diagnosis received [e.g., (37, 60)].

#### ASQ

The ASQ assesses Communication, Gross Motor, Fine Motor, Problem Solving, and Personal-Social skills in children aged 0–5 ½ years (82). Of forty-three studies looking at the psychometric properties of the tool, twenty-three examined the accuracy of the ASQ (32, 36, 41, 42, 44–51, 53, 57, 60–62, 65, 66, 68–71), and one study evaluated the Infant/Child Monitoring Questionnaire, which was surpassed by the ASQ (33). Seven studies were undertaken in the USA, five in Canada, two in China, two in Norway, one in Australia, one in Chile, one in Japan, one in Korea, one in The Netherlands, one in Portugal, and one in Spain. Ten studies recruited participants from health care settings (32, 36, 44–48, 51, 66, 70), nine from a community and/or health setting (33, 41, 42, 49, 50, 53, 57, 60, 61), two from preschools (69, 71), one from a child development support program (68), one used data from a national longitudinal survey of children and youth (65), and one used population data (62) (total *N* = 37,144). Together the results showed that the sensitivity of the tool ranged from 22% (32) to 100% (36, 60) while specificity ranged from 39% (46) to 92% (50). Most studies, however reported that the sensitivity and/or specificity of the ASQ fell within the moderate range (the sensitivity and specificity ranges referenced were: High = >90%, Moderate = 71%–90%, Low = ≤70%) (32, 36, 44, 49, 50, 60). Furthermore, most studies assessing accuracy indicated a low risk of bias on the QUADAS-2 (Table 1).

#### PEDS

The PEDS is a developmental screener consisting of ten questions that assesses for global/cognitive, expressive language and articulation, receptive language, fine and gross motor, behaviour, self-help, socialisation, and academic concerns in children aged 0–8 years (83). Seven studies evaluated the psychometric properties of the PEDS (30, 44, 48, 63, 69, 70, 80). Four studies (total *N* = 7,155) were undertaken in the USA, one in Canada, one in Spain, and one in Singapore. Four studies recruited participants from primary care centres (44, 48, 70, 80), one from community and health care settings (30), one from childcare and kindergarten centres (63), and one from pre-primary school (69). Overall results indicated the measure to have moderate sensitivity (74%–83%) (30, 44) and low to moderate specificity (64%–84%) (30, 44). One study also found the measure to have high internal consistency and test-retest reliability, and moderate to high interrater reliability (when completed by parents and professionals) (30).

#### ESSENCE-Q

The ESSENCE-Q is a developmental screener comprised of twelve items that assess development in children under the age of six (37, 38, 81). All three studies evaluating the ESSENCE-Q were undertaken in Japan by the same team (37, 38, 81). Participants (total *N* = 381) were recruited from primary (i.e., public health clinic) and secondary (i.e., developmental clinic) health care settings. Together results showed the screener to have moderate to high sensitivity (ranging between 78% and 94%) when completed by mothers, nurses and psychologists, low specificity when completed by mothers (59%), and low to moderate specificity when completed by psychologists and nurses (ranging between 53% and 77%).

#### SWYC

The SWYC consists of short answer questions relating to child development, child behaviour, and family risk factors (e.g., depression, substance abuse) completed by caregivers of children aged 1–65 months (84). Of the three identified SWYC studies, two evaluated the psychometric properties of the SWYC (48, 73). Both studies evaluating the psychometric properties of the SWYC were undertaken in the USA by the same team, and participants were recruited from primary care settings (total *N* = 14,571). Results from Sheldrick, Schlichting (73) indicated that a higher percentage of children were reported to pass milestones by the age at which the Centres for Disease Control and Prevention (CDC) guidelines indicated that “most children pass” certain milestones and a greater percentage of children were reported to pass milestones by the age at which the CDC stated that parents should “act early”. The second study found that the screener had moderate specificity (70.8%–89%) and moderate sensitivity but only for severe delays (73.3%) (48).

#### Denver II

The Denver II is a clinician-administered developmental screening test that is comprised of 125 items which assess general areas of development in children aged 0–6 years (85). Two studies assessed the accuracy of the Denver II, both studies were undertaken in the USA by the same team and recruited participants (total *N* = 193) from childcare centres (34, 35). Together results indicated the measure to have moderate sensitivity (>80%) and low specificity (<50%).

#### BINS

The BINS is an 11–13 item (depending on age at administration) developmental screener for children aged 3–24 months (86). The overall score is classified as low, moderate or high and reflects a child's risk of developmental delay or neurological impairment (86). The accuracy of the BINS was evaluated in one study undertaken in the USA and recruited participants (*N* = 106) from hospitals (39). Results showed the measure to have low sensitivity (ranging from 0% to 40%) but high specificity (ranging from 66% to 100%).

#### CDI

The CDI is a revised version of the MCDI. It is a parent-completed screener that measures eight areas of development (social, self-help, gross motor, fine motor, expressive and receptive language, letters and numbers) in children aged 15 months to 6 years (87). One study was found that evaluated the CDI (46), and one evaluated the MCDI (40). An additional study was found that assessed the MPI—a shortened version of the MCDI used with preschool-aged children (72). Given that the CDI surpasses the MCDI, only the CDI results were interpreted. The CDI study was undertaken in Canada and recruited participants from primary care settings (*N* = 246). Results indicated that the measure had low sensitivity (50%), low positive predictive validity (50%), moderate specificity (86%), and moderate negative predictive validity (86%).

#### CREDI

The CREDI is a newly developed parent-completed child development screener for children aged 0–35 months (88). One study evaluated the psychometric properties of the CREDI (43). The study was undertaken in China and recruited participants from urban and rural communities (*N* = 946). Results indicated that the measure had good internal consistency reliability (Cronbach's alpha = .92–.97) reliability and high concurrent validity with the Bayley Scale of Infant and Toddler Development-III.

#### DSQ

The DSQ is a parent-completed, computer-based child-screening questionnaire designed to evaluate developmental risk in children aged 1–6 years (64). One study evaluated the accuracy of the DSQ (64). The study was undertaken in Singapore and recruited a community sample (*N* = 506). Results showed the measure to have high specificity (97%) and no data was provided on sensitivity.

#### EYCI

The EYCI is a newly developed, parent-completed screener designed to assess developmental concerns in children aged 18 months to 6 years. The measure is comprised of 11 items which examine 10 domains and is available both in paper format and electronically (31). One study assessed the accuracy of the EYCI. The study was undertaken in Canada, recruited participants (*N* = 246) from childcare settings, and had both parents and educators complete the measure. Results showed the measure to have moderate sensitivity (86%) and specificity (82%) when completed by parents. Agreement between parents and educators was low (Rho > 0.30).

#### SGS-II

The SGS-II is a developmental screening tool targeted at assessing developmental trajectories in children aged 0–5 years. The tool is an adapted version of the National Childhood Encephalopathy Study tool (89) and is comprised of four subscales (locomotor, language, personal-social, and fine motor) which assesses ten different skill areas (52). One study assessed the accuracy of the SGS-II (52). The study was undertaken in North Wales and families (*N* = 39) were recruited from nurseries and nursery schools. Results indicated that the measure had high sensitivity (100%; except for the locomotor subscale which had low sensitivity) and specificity (100%) for children aged 0–24 months and moderate to high sensitivity (50%–100%) and moderate to high specificity (85%–91%; except for the locomotor subscale which had low specificity) for children aged 25–52 months.

#### TBCS

The TBCS is a developmental screener designed to assess gross motor, fine motor, language, and social skills in children at 6, 18, and 36 months (67). A pilot study was undertaken in China to assess the screener (67). The study recruited a community sample of participants (*N* = 1,783). Results indicated that the measure had good predictive validity with the 6-month assessment results predicting the 18-month results, and the 18-month results predicting the 36-month results.

#### Taipei II

The Taipei II provides a checklist for thirteen age groups from 4 months to 6 years, which assesses fine motor, gross motor, language/communication, and emotion/social areas (75). One study evaluated the accuracy of the Taipei II (75). The study, which was undertaken in Taiwan (China) and recruited participants (*N* = 390) from a public health centre, evaluated the psychometric properties of the digital version of the measure compared to the original text version. Results indicated excellent agreement between the two versions of the measure as well as moderate to high reliability.

### Knowledge, acceptability, and feasibility of universal developmental screening tool

Nine studies were identified that explored knowledge, acceptability, and/or feasibility of administering universal developmental surveillance and screening tools (54–56, 58, 74, 76–79). Five studies evaluated the LTSAE (55, 74, 77–79), two evaluated the PEDS (54, 58), one the ASQ (54), one the Taipei II (76), and one the SWYC (56). Six were undertaken in the USA, two in Australia, and one in China.

### Developmental surveillance tools

#### LTSAE

The LTSAE program is aimed at improving identification of children with developmental delays (90). The program includes a short developmental checklist targeted at children aged 2 months to 5 years as well as educational materials regarding child development for parents and service providers (90). In 2022, following a review of literature, the Centers for Disease Control and Prevention updated the LTSAE (91). Eleven criteria were developed to assess milestones, including that the milestones are indicated at an age that most children (75%) would expect to demonstrate mastery of the milestone and that the milestones are easy for families from different social, cultural and ethnic groups to observe (91). All five studies examining the knowledge, acceptability, and/or feasibility of administering of the LTSAE were undertaken in the USA (55, 74, 77–79). Two studies recruited participants from Head Start centres (74, 79), two from child care centres (55, 78), and one from well-child visits (77). Together, results showed that the LTSAE materials increased parental knowledge and awareness of developmental milestones. One study, however, noted that while parents found LTSAE materials appealing, they were unaware of how to act early or why acting early was important. The two studies undertaken at Head Start centres (74, 79) also found that participation in the LTSAE program improved parental engagement with developmental monitoring, improved communication between parents and staff members, and helped build rapport between parents and staff members. Abercrombie, Pann (74) also showed that most Head Start staff in their study found the materials easy to integrate into work with their families and did not experience any barriers to doing so, indicating that it is feasible to incorporate LTSAE materials into the Head Start program. Of the small percentage of Head Start staff (11%) that did identify barriers, the most common barriers included lack of time, conflicting demands, parents being unreceptive to materials, and low parental literacy.

### Developmental screening tools

#### ASQ

Only one study was identified which examined the knowledge and acceptability of administering the ASQ. The study was undertaken by Garg, Ha (54), and looked at 37 Australian health professionals’ [three nurse managers, one Out-of-Home Care coordinator (social worker), two general practitioner practice nurses, five child and family health nurses, six general practitioners, seven paediatricians and one senior child health medical officer] knowledge and acceptability of the ASQ-3 (and the PEDS). Results indicated that most health professionals were aware of the benefits associated with developmental screening tools though several barriers were identified that might prevent them from utilising screening tools. These barriers included the availability of the screening tool in languages other than English, limited knowledge of the screening tool, and concerns regarding the specificity of the screening tool. Some Paediatricians and General Practitioners also noted that they relied on their clinical judgment rather than screening tools to identify developmental delays.

#### PEDS

Two Australian studies looked at the knowledge and acceptability of the PEDS (54, 58). One study included health professionals recruited from primary care settings (54) and the other included parents (*N* = 262) recruited from childcare centres and kindergarten (58). As mentioned above (in the ASQ section), most health professionals were aware of the benefits and barriers associated with screening tools. Parents indicated that the measure was easy to complete and felt that the information would be helpful to health professionals.

#### Taipei II

Cheng, Chang (76) evaluated 118 caregivers’ acceptability and utility of the multimedia version of the Taipei II. Results showed that the measure was easily accessible and that 98% of participants preferred the multimedia version of the measure to the paper version, indicating strong support for the acceptability of the tool.

#### SWYC

Berger-Jenkins, Monk (56) evaluated the feasibility of administering an adapted SWYC together with a behavioural screener in a busy urban medical practice (*N* = 349 parents). Results showed that screening rates ranged between 5% and 90%. Screening tables were introduced when barriers, such as running out of photocopies and not knowing which patients to give screeners to, were identified. After the introduction of screening tables, screening rates reached as high as 90%.

### Action taken following positive screening

Nine studies provided information on action taken following developmental screening (38, 40, 44, 56, 58, 60, 69, 74, 81). Two were undertaken in Canada, two in Japan, three in the USA, one in Australia, and one in Spain.

Studies either indicated that information regarding test scores was provided to parents who were instructed to seek follow-up assessments or that participants were referred for follow-up assessments. Only two studies, however, conducted follow-ups to determine rates of referral uptake. Berger-Jenkins, Monk (56) noted that following screening using the SWYC (and a behavioural screener) approximately 80% of participants followed up with their primary medical doctor and 50% completed referrals to a clinic social worker. Abercrombie, Pann (74) found that 28% of their participants had child development concerns. Of those, 51% reported that LTSAE materials helped identify concerns. Furthermore, 52% of participants who reported speaking to a professional noted that the materials were very helpful, and 26% found the materials helpful, when speaking with their doctors. The reported outcomes of these visits included 28% of parents receiving additional information about their concerns, 28% being referred to another professional, 25% receiving current help with their concerns, 16% reported that their child received a diagnosis, and 3% noted that their doctor did not identify a delay.

## Discussion

This review has described and synthesized the available evidence on the psychometric properties; knowledge, feasibility, and acceptability; and evidence of outcomes of developmental screening and surveillance implementation in high income countries. Fifty-two articles were evaluated to ascertain evidence regarding the psychometric properties of identified tools; knowledge, acceptability, and feasibility of identified tools; and actions taken following positive screens.

The first finding was that a number of studies assessing the psychometric properties, including accuracy, of developmental screening tools have been published, however, the majority of identified literature evaluated the ASQ (*n* = 23). Overall, for screening tools which had more than one study undertaken (e.g., ASQ, PEDS), findings regarding the accuracy of the tool were highly variable, particularly so when measures were administered in different countries or with different populations. Given these findings it is critical that care is taken to ensure that a measure has been validated for use with a specific population before it is administered with that population to avoid over- or under-recognition of developmental disorders (92). Furthermore, the operationalisation of sensitivity and specificity differed across studies with some studies comparing screener scores to outcomes on standardised developmental measures while others compared screener scores to a diagnosis of a developmental condition. Given that the aim of screeners is to identify developmental conditions; that diagnosis of a developmental conditions requires more than a positive score on a standardised developmental measure; and that most studies looked at the correlation between screener scores and developmental measure outcomes to ascertain sensitivity and specificity, more research needs to be undertaken to determine the sensitivity and specificity of measures in regard to receiving a diagnosis of a developmental condition.

Second, several studies assessing the knowledge relating to, and acceptability and feasibility of, using screening and surveillance tools were also identified, with most identified literature examining the LTSAE (*n *= 5). Results provided support for each measure's acceptability and/or feasibility. The literature was however limited by only a small number of studies examining each tool. More research examining the acceptability and feasibility of all identified screening tools is recommended. This would increase understanding regarding which measures are well received by target populations and more likely to be completed (93).

Third, a few studies noted that action was taken following positive developmental screens, though, only two studies (56, 74) conducted follow-up assessments to describe these actions. Without assessing outcomes of screening, it is difficult to determine the benefits and risks associated with screening. Commonly cited benefits associated with early screening often include early diagnosis and access to early intervention (94, 95). Early intervention has been found to support the development of core (e.g., communication) and related (e.g., play) areas of child development (96). In contrast, risks frequently associated with screening include receiving a false positive screen and the time, effort, and anxiety associated with further testing (97). More research is therefore needed to determine whether the benefits of screening for developmental disorders outweigh the risks.

### Clinical and policy implications

This review identified a variety of available universal developmental screeners. While literature on the acceptability and feasibility of screeners indicated that the administration of most screeners was feasible and acceptable, the accuracy of some screeners varied across studies. Furthermore, with the exception of the ASQ, PEDS, and LTSAE, most screeners were evaluated in three or less studies (with the majority only being evaluated in one study). If developmental screeners are to be implemented universally (e.g., national screening programs), it is necessary to consider the research evidence available on that screener. However, it is also necessary to examine the accuracy, acceptability, and feasibility of the chose screener with the population it is intended to be used with and ensure that they are able to detect developmental delays in target populations (98), before widespread implementation occurs.

### Strengths and limitations

Strengths of this review included the use of systematic review strategy with broad inclusion criteria, and that reviewing of all included studies and completion risk of bias assessments was undertaken by two independent reviewers. The review also had several limitations. First, searches were restricted to studies written in the English language, reducing the generalizability of findings. Second, the quality assessment indicated that several of the diagnostic accuracy studies did not report information on whether results of the knowledge standard tests (e.g., secondary, or diagnostic assessment) were interpreted without knowledge of the index test (e.g., screener) results suggesting an increased risk of bias. Third, the psychometric performance of screening tools can be influenced by cultural variations in behavioural expectations and developmental milestones across diverse populations. We did not thoroughly assess cultural variation, as this was beyond the scope of our study. Fourth, given that the focus of the review was on high income countries, the results cannot be generalized to low- and middle-income countries. Fifth, studies that fit the inclusion criteria but did not segregate data based on population or country were excluded. This may have impacted the study outcomes.

## Conclusion

To conclude, developmental screeners have been considered useful tools for early identification difficulties. This review found that a variety of developmental screening/surveillance tools are available, however, the majority of identified studies have been undertaken on only a small number of available tools (e.g., ASQ, PEDS, LTSAE), with most screeners only being evaluated in one study. For screeners which have been evaluated in more than one study, the accuracy of the tools was found to vary across countries and populations. It is necessary to ensure that a screener is accurate, feasible, and acceptable to be administered with a certain population before it is used with that population. Without evidence supporting the accuracy of a screener for use with a certain population, the use of the screener may result in false positive screens and the associated negative consequences. Thus, if universal implementation of a developmental screener is to occur, the accuracy, acceptability, and feasibility associated with administrating that screener will need careful consideration before the screener is implemented.

## Data Availability

The original contributions presented in the study are included in the article/**Supplementary Material**, further inquiries can be directed to the corresponding author.
