# Supplementary material for: Developmental screening tools for identification of children with developmental difficulties in high-income countries: a systematic review
Source: Front Child Adolesc Psychiatry. 2023 Jul 6;2:1074004. doi: 10.3389/frcha.2023.1074004 (PMC11732054; doi:10.3389/frcha.2023.1074004)
Supplement: Supplementary file 1 [file Table1.docx]

**Supplementary Tables**

**Supplementary Table 1.**

Search strategy and search terms

| Database | Time frame | Results |
| --- | --- | --- |
| PsychInfo | Pre 2014 | [(noft("child" OR "infant*" OR "baby" OR "preschool") AND noft("milestone*" AND "surveillance" OR "screening tool*" OR "screening measure*" OR "screening assessment*")) AND rtype.exact("Journal Article") AND la.exact("English") AND age.exact("Neonatal (birth-1 Mo)" OR "Infancy (2-23 Mo)" OR "Preschool Age (2-5 Yrs)") AND po.exact("human") AND PEER(yes) AND pd(<20140101)](https://www.proquest.com/myresearch/savedsearches.checkdbssearchlink:rerunsearch/2190435/SavedSearches?site=psycinfo&t:ac=SavedSearches) |
| PsychInfo | Post 2014 | noft( “child” OR “infant*” OR “baby” OR “preschool” ) AND noft(“milestone*” AND “surveillance” OR “screening tool*” OR “screening measure*” OR “screening assessment*” ) |
| Embase | Pre 2014 | \| Embase Classic+Embase <1947 to 2022 July 12> \| \| \| \| --- \| --- \| --- \| \|  \|  \|  \| \| 1 \| child/ \| 2,219,643 \| \| 2 \| infant/ \| 770,967 \| \| 3 \| baby/ \| 23,662 \| \| 4 \| preschool.mp. \| 686,272 \| \| 5 \| 1 or 2 or 3 or 4 \| 2739697 \| \| 6 \| milestone.mp. \| 8935 \| \| 7 \| surveillance.mp. \| 351129 \| \| 8 \| screening tool.mp. \| 34293 \| \| 9 \| screening measure.mp. \| 1410 \| \| 10 \| screening assessment.mp. \| 1532 \| \| 11 \| 7 or 8 or 9 or 10 \| 387434 \| \| 12 \| 5 and 6 and 11 \| 43 \| \| 13 \| limit 12 to (human and english language and yr="1883 - 2013" and journal and preschool child <1 to 6 years>) \| 4 \| \| 14 \| 12 and 13 \| 4 \| |
| Embase | Post 2014 | Database: Embase Classic+Embase <1947 to 2022 May 17> Search Strategy: -------------------------------------------------------------------------------- 1 child/ (2201344) 2 infant/ (766077) 3 baby/ (23741) 4 preschool.mp. [mp=title, abstract, heading word, drug trade name, original title, device manufacturer, drug manufacturer, device trade name, keyword heading word, floating subheading word, candidate term word] (682234) 5 1 or 2 or 3 or 4 (2719009) 6 milestone.mp. [mp=title, abstract, heading word, drug trade name, original title, device manufacturer, drug manufacturer, device trade name, keyword heading word, floating subheading word, candidate term word] (8783) 7 surveillance.mp. [mp=title, abstract, heading word, drug trade name, original title, device manufacturer, drug manufacturer, device trade name, keyword heading word, floating subheading word, candidate term word] (346526) 8 screening tool.mp. [mp=title, abstract, heading word, drug trade name, original title, device manufacturer, drug manufacturer, device trade name, keyword heading word, floating subheading word, candidate term word] (33792) 9 screening measure.mp. [mp=title, abstract, heading word, drug trade name, original title, device manufacturer, drug manufacturer, device trade name, keyword heading word, floating subheading word, candidate term word] (1393) 10 screening assessment.mp. [mp=title, abstract, heading word, drug trade name, original title, device manufacturer, drug manufacturer, device trade name, keyword heading word, floating subheading word, candidate term word] (1517) 11 7 or 8 or 9 or 10 (382313) 12 5 and 6 and 11 (40) 13 limit 12 to (human and english language and yr="2014 -Current" and journal and preschool child <1 to 6 years>) (8) 14 12 and 13 (8) |
| PubMed | Pre 2014 | Search: **("child") OR ("infant") OR ("baby") OR ("preschool") AND ("milestone") AND (surveillance) OR ("screening tool*") OR ("screening measure") OR ("screening assessment")** Filters: **Clinical Study, Clinical Trial, Observational Study, Randomized Controlled Trial, Humans, English, Infant: birth-23 months, Infant: 1-23 months, Preschool Child: 2-5 years, from 1989/1/1 - 2013/12/31**((("child"[All Fields] OR "infant"[All Fields] OR "baby"[All Fields] OR "preschool"[All Fields]) AND "milestone"[All Fields] AND ("epidemiology"[MeSH Subheading] OR "epidemiology"[All Fields] OR "surveillance"[All Fields] OR "epidemiology"[MeSH Terms] OR "surveilance"[All Fields] OR "surveillances"[All Fields] OR "surveilled"[All Fields] OR "surveillence"[All Fields])) OR "screening tool*"[All Fields] OR "screening measure"[All Fields] OR "screening assessment"[All Fields]) AND ((clinicalstudy[Filter] OR clinicaltrial[Filter] OR observationalstudy[Filter] OR randomizedcontrolledtrial[Filter]) AND (humans[Filter]) AND (1989/1/1:2013/12/31[pdat]) AND (english[Filter]) AND (allinfant[Filter] OR infant[Filter] OR preschoolchild[Filter])) |
| PubMed | Post 2014 | Search: ("child") OR ("infant") OR ("baby") OR ("preschool") AND ("milestone") AND (surveillance) OR ("screening tool*") OR ("screening measure") OR ("screening assessment") Filters: Clinical Study, Clinical Trial, Observational Study, Randomized Controlled Trial, Humans, English, Infant: birth-23 months, Infant: 1-23 months, Preschool Child: 2-5 years, from 2014/1/1 - 2022/5/18 ((("child"[All Fields] OR "infant"[All Fields] OR "baby"[All Fields] OR "preschool"[All Fields]) AND "milestone"[All Fields] AND ("epidemiology"[MeSH Subheading] OR "epidemiology"[All Fields] OR "surveillance"[All Fields] OR "epidemiology"[MeSH Terms] OR "surveilance"[All Fields] OR "surveillances"[All Fields] OR "surveilled"[All Fields] OR "surveillence"[All Fields])) OR "screening tool*"[All Fields] OR "screening measure"[All Fields] OR "screening assessment"[All Fields]) AND ((clinicalstudy[Filter] OR clinicaltrial[Filter] OR observationalstudy[Filter] OR randomizedcontrolledtrial[Filter]) AND (humans[Filter]) AND (2014/1/1:2022/5/18[pdat]) AND (english[Filter]) AND (allinfant[Filter] OR infant[Filter] OR preschoolchild[Filter])) |

Note: Database searchers were divided into searchers pre- and post-2014. The review was initially intended to be completed on articles available post-2014. The criteria for the review were then revised and searches on articles available pre-2014 were undertaken.

**Supplementary Table 2.**

Definitions of low, lower middle, upper middle, and high income countries.

| **Classification as per world bank** | **Definition** |
| --- | --- |
| Low income | Low-income economies are those with Gross National Income (GNI) per capita, calculated using the *World Bank Atlas method*, of $1,085 or less in 2021 |
| Lower middle income | Lower-middle-income economies are those with a GNI per capita, calculated using the *World Bank Atlas method*, of between $10865 and $4,255 |
| Upper middle income | Upper-middle-income economies those with a GNI per capita, calculated using the *World Bank Atlas method*, between $4,256 and $13,205 |
| High-income | High-income economies are those with a GNI per capita, calculated using the *World Bank Atlas method*, of $13,205 or more. |

Note: This definition has been sourced from the World Bank and can be accessed via ([World Bank Country and Lending Groups – World Bank Data Help Desk](https://datahelpdesk.worldbank.org/knowledgebase/articles/906519-world-bank-country-and-lending-groups)).

**Supplementary Table 3.**

High income countries list (2021)

| Andorra | Greece | Poland |
| --- | --- | --- |
| Antigua and Barbuda | Greenland | Portugal |
| Aruba | Guam | Puerto Rico |
| Australia | Hong Kong SAR, China | Qatar |
| Austria | Hungary | Romania |
| Bahamas, The | Iceland | San Marino |
| Bahrain | Ireland | Saudi Arabia |
| Barbados | Isle of Man | Seychelles |
| Belgium | Israel | Singapore |
| Bermuda | Italy | Sint Maarten (Dutch part) |
| British Virgin Islands | Japan | Slovak Republic |
| Brunei Darussalam | Korea, Rep. | Slovenia |
| Canada | Kuwait | Spain |
| Cayman Islands | Latvia | St. Kitts and Nevis |
| Channel Islands | Liechtenstein | St. Martin (French part) |
| Chile | Lithuania | Sweden |
| Croatia | Luxembourg | Switzerland |
| Curaçao | Macao SAR, China | Taiwan, China |
| Cyprus | Malta | Trinidad and Tobago |
| Czech Republic | Monaco | Turks and Caicos Islands |
| Denmark | Nauru | United Arab Emirates |
| Estonia | Netherlands | United Kingdom |
| Faroe Islands | New Caledonia | United States |
| Finland | New Zealand | Uruguay |
| France | Northern Mariana Islands | Virgin Islands (U.S.) |
| French Polynesia | Norway |  |
| Germany | Oman |  |

Note: This list has been sourced from the World Bank and can be accessed via ([World Bank Country and Lending Groups – World Bank Data Help Desk](https://datahelpdesk.worldbank.org/knowledgebase/articles/906519-world-bank-country-and-lending-groups)).
